# The Effect of Plasma Pretreatment and Cross-Linking Degree on the Physical and Antimicrobial Properties of Nisin-Coated PVA Films

**DOI:** 10.3390/ma11081451

**Published:** 2018-08-16

**Authors:** Zuzana Kolarova Raskova, Pavel Stahel, Jana Sedlarikova, Lenka Musilova, Monika Stupavska, Marian Lehocky

**Affiliations:** 1 Centre of polymer systems, Tomas Bata University, Trida Tomase Bati 5678, 76001 Zlin, Czech Republic; sedlarikova@utb.cz (J.S.); musilova@utb.cz (L.M.); lehocky@utb.cz (M.L.); 2 Department of Physical Electronics, Faculty of Science, Masaryk University, Kotlarska 267/2, 63711 Brno, Czech Republic; pstahel@physics.muni.cz (P.S.); 119414@mail.muni.cz (M.S.); 3 Department of Fat, Surfactants and Cosmetics Technology, Faculty of Technology, Tomas Bata University, Vavrečkova 275, 76001 Zlin, Czech Republic

**Keywords:** antimicrobial film, nisin, physical properties, plasma treatment polyvinyl alcohol, surface characterization

## Abstract

Stable antimicrobial nisin layers were prepared on the carrying medium-polyvinyl alcohol (PVA) films, crosslinked by glutaric acid. Surface plasma dielectric coplanar surface barrier discharge (DCSBD) modification of polyvinyl alcohol was used to improve the hydrophilic properties and to provide better adhesion of biologically active peptide-nisin to the polymer. The surface modification of films was studied in correlation to their cross-linking degree. Nisin was attached directly from the salt solution of the commercial product. In order to achieve a stable layer, the initial nisin concentration and the following release were investigated using chromatographic methods. The uniformity and stability of the layers was evaluated by means of zeta potential measurements, and for the surface changes of hydrophilic character, the water contact angle measurements were provided. The nisin long-term stability on the PVA films was confirmed by tricine polyacrylamide gel electrophoresis (SDS-PAGE) and by antimicrobial assay. It was found that PVA can serve as a suitable carrying medium for nisin with tunable properties by plasma treatment and crosslinking degree.

## 1. Introduction

Antimicrobial surfaces and packaging have been receiving increasing attention over the last few decades. Except antimicrobial properties, high levels of biodegradability and biocompatibility are important attributes of polymer materials that are suitable for medical and pharmaceutical applications and the cosmetic and food industries. The polymeric drug delivery systems with controlled release and increased solubility of the drug are investigated. Drugs or antimicrobial agents can be incorporated directly into polymers or they are attached via the side chains often with peptides [1,2,3,4,5]. 

Among environmentally favorable polymers, a synthetic biodegradable polyvinyl alcohol (PVA) possessing excellent mechanical properties is one of the most important representatives. PVA became attractive also due to its inherent hydrophilicity, large swelling capacity, low cost, and simplicity of use. Its biodegradability in various microbial environments has been reported [6,7,8,9,10]. 

PVA provides many potential functional groups for the adhesion of antimicrobial and preserving agents, among which the natural based ones are the most promising. 

In particular, the proteins have been extensively investigated as a coating material. However, the immobilization of proteins is often associated with their tendency to lose their biological activity [6,11,12,13,14]. The use of peptides offers a solution to this problem. The peptides can be obtained as a part of protein after their digestion or as lantibiotics/bacteriocins [7,8,9,10]. Nisin is an amphiphilic peptide (bacteriocin) with low molecular weight (approximately 3.4 kDa) that is an effective inhibitor of Gram-positive bacterial strains [9,10,13,14,15,16], which are produced by lactic acid bacterial strain *Lactococcus lactis* during the fermentation process. Nisin has been studied as a promising preserving agent since the 1950s [17,18]. Moreover, nisin (and another bacteriocins) provide sufficient antimicrobial activity against bacterial strains that are resistant to conventional drugs. The antitumor activity of nisin is reported in several studies [19,20,21]. 

Antimicrobial properties of the films are based on peptide immobilization or incorporation and release. For the bioactivity of film/foils, peptide stability is very important. Many types of physical and chemical treatments have been employed to obtain uniform and stable surface treatment [18,19,20,21,22]. Atmospheric plasma surface activation can offer suitable functional groups for the binding of bioactive substances [21,23,24]. In many studies, the suitability of Dielectric Coplanar Surface Barrier Discharge (DCSBD) treatment was evaluated [25]. This discharge type was investigated with regard to industrial use, and the importance of dielectric barrier discharges for material processing has been continually increasing. This follows from the current trends in industrial applications, in which an effort to replace low-pressure plasma processing by the atmospheric-pressure systems can be observed. This discharge has been successfully used for polymer surface activation [24,26]. It operates in a non-thermal, uniform, glow plasma regime. Its advantage is manifold symmetry; therefore, it can be used also for large area surface modification [25,27]. Some studies have shown the significant effect of DCSBD on the selective functionalization of polymers; however, the DCSBD treatment on biodegradable PVA based polymers with regard to peptides adhesion on their surfaces, as well as on the peptide layer stability, was not reported before. The main advantage of choosing air as the process gas is that it is naturally present in the industrial production lines [26]. Contrary to other dielectric barrier discharges, the DCSBD is capable of generating a macroscopically uniform plasma layer with high power density and energetic efficiency when operated in air [27,28,29]. This enables fast and homogeneous in-line plasma treatment of materials at high-speeds.

The methods for setting up antimicrobial polymeric surfaces are still in developmental stages, and understanding the structure-function relationship of the material is important to the design and control of delivery systems. Although the surface modification for peptide and protein adhesion as well as the mechanism of adhesion were studied in many papers [30,31,32,33,34,35,36,37,38,39,40,41,42,43,44], the nisin stability, and thus its long-term antimicrobial effect, was not sufficiently reported. Therefore, the objective of this study is to evaluate the plasma treatment effect on the physico-chemical properties of PVA films as potential transport system for nisin. Also, nisin stability, its release from the surface, and the antibacterial properties of modified PVA films were investigated in correlation with crosslinking degree.

## 2. Materials and Methods

### 2.1. Materials

Polyvinyl alcohol (PVA, Mowiol 8-88), glutaric acid (GA), Polyethylene glycol (PEG) 2050, phosphate-buffered saline (PBS, pH 4.5), methanol, lactose, trifluoroacetic acid (TFAA), trichloracetic acid (TCA), formic acid, potassium chloride (KCl), ammonium bicarbonate (NH_4_HCO_3_), acrylamide, *N*,*N*′-methylene bisacrylamide, Coomasie brilliant blue R250, glutaraldehyde, molecular weight (MW) markers, nisin from lactococcus lactis standard (2.5%, balance sodium chloride and denatured milk solids) and bovine serum albumin (BSA), and Tween 80 were purchased from Sigma-Aldrich (St. Louis, MO, USA).

Tryptone soya agar (TSA), Nutrient Broth, MRS broth, and Mueller-Hinton agar (MHA) were obtained from HiMedia (Mumbai, India), bacterial strain *Staphylococcus aureus* CCM 4516 was purchased from Czech Collection of Microorganisms (Masaryk University in Brno, Czech Republic). 

### 2.2. PVA Films Preparation

Prior to experiments, PVA films were prepared using solvent cast technique (10 wt. % of PVA, at 85 °C) from aqueous/HCl (0.02 M) solution. Glutaric acid (GA) was used as the crosslinking agent at different concentrations to obtain following crosslinking degrees: 0%, 5%, 10%, 20%, and 40% [3,45].

In most experiments, the PVA was prepared on microscopy glass (Fischer Sci, Pardubice, Czech Republic diameter: 26 × 26 × 1.1 mm). However, for the purpose of long-time electrokinetic measurement (titration curves), it was necessary to prepare PVA films on polyethylene (PE) foil substrate that prevented the PVA foil twisting under high pressure of electrolyte. The PE matrix was activated by 10 s plasma treatment [6,9,11] before the PVA dip-coating to improve the adhesion and homogeneity.

### 2.3. PVA Activation and Nisin Application

It is well known [2,9,13,16] that adhesion of peptides/nisin on many polymers is rather unsatisfactory, and so additional polymer activation step was carried out.

The PVA film surface was activated by two types of Dielectric Coplanar Surface Barrier Discharge (DCSBD), RPS600 and RPS 40 systems (Roplass s.r.o., Brno, Czech Republic). DCSBD consists of two comb-like electrodes printed on Al_2_O_3_ dielectric plate [15,25,26] in ambient air under the following condition sets: (1) power generator 14 kHz, surface power density 2 W/cm^2^ (RPS 40) and (2) power generator 25 kHz, power density 7 W/cm^2^ (RPS600). The treatment time was 10 s. The distance between the sample surface and plasma layer was 0.3 mm. The process was carried out in the dynamic regime to obtain homogeneous surface treatment.

On the surface of PVA films, 400 µL of commercial nisin solution was applied (nisin was dissolved in phosphate saline solution, the pH value was set at 4.5, and the nisin concentration was 525 µg/mL). It means that theoretical amount of nisin on the surface was 0.31 µg/mm^2^. The samples were incubated for 20 min at room temperature, and then the surface was dried and shortly rinsed off by demineralized sterilized water and dried again.

As the finishing step, all samples were plasma-treated for 10 s to improve stability and sterility [19,20,21,23].

Just for comparison, a set of samples without the PVA plasma activation was prepared. They proved weak adhesion, homogeneity, and stability, which made them generally not applicable.

### 2.4. Release and Stability Analysis of Nisin

Nisin layers behavior in water (water resistance) was tested via nisin release from the prepared coatings that was carried out at preselected time intervals, while the PVA films were immersed into physiological solution for 1 month. The nisin concentrations in the eluate were analysed by reverse-phase HPLC using a C-18 column (5 µm, 4.6 × 250 mm, Waters) with pre-column Reprosil 100 C18 (5 µm, 50 × 4 mm, Watrex eluted with water/0.05% (*v*/*v*) TFA (eluent A) and acetonitrile/0.05% (*v*/*v*) TFA (eluent B) (gradient: 0–5 min, 20% eluent A; 5–20 min from 20% eluent A, to 80% eluent A) with UV detection at 220 nm. The injection volume was 25 µL, and the flow rate was of 0.6 ml/min. The amount of nisin was calculated by means of a calibration curve, when calibration solutions were prepared from 0.2 M filtered (the syringe filter with pore size 0.45 µm) solution.

The nisin release profile was tested also during long-term stability testing (90 days of storage in conditioned chamber at 25 °C and relative humidity (RH) of 54% of films). Simultaneously, the stability of released nisin was studied electrochemically using Tricine SDS-PAGE at 16% and 4% separating, and stacking acrylamide gel containing 3% bis-acrylamide, proteins, and peptides were visualized by staining solution: methanol/acetic acid/ water/Coomassie Blue G250 and by silver staining. After electrophoresis, the bands from the electrophoregrams were cut, and, after spot destaining, the peptide was extracted by 5% formic acid/acetonitrile (1:2) solution, and the nisin concentrations were analyzed by means of UV-VIS spectrophotometry with ELISA reader at a wavelength of 595 nm.

Besides, total nitrogen content eluted from the surface after films immersion (3 h into physiological solution) was measured by TOC/TN analyzer (TNM-L, Shimadzu, autosampler ASI-L, Shimadzu Corporation, Japan). Calibration curve was created using potassium nitrate solution in ultrapure water.

The results presented further are the average values from at least three measurements. Standard deviation value was always up to 10% of average value. 

### 2.5. Antimicrobial Assays

To study the long-term stability of nisin coatings, the as-prepared samples were stored in conditioned chamber at 25 °C and relative humidity of 54%, while the measurements were performed also by antimicrobial testing, initially with freshly made coatings and with the coatings after their storage.

Ageing of these layers was monitored as a decrease of antibacterial activity of both released nisin and nisin surface by means of two types of testing.

Nisin in water activity was determined by agar diffusion method [42,43] using *Staphylococcus aureus* as the nisin sensitive microorganism. Nisin layers were immersed into demineralized sterile water, and eluate was used for testing. An aliquot of bacterial culture (10^8^ cfu/mL) was applied with a sterile swab on the surface of the Mueller Hinton agar plate. The eluate (3 h into physiological solution) from the nisin coated samples was transferred into the bored wells of 8 mm in diameter (120 µl/well) and incubated at 35 °C for 24 h. Control samples were prepared as series of nisin solutions in demineralized and sterilized water, starting with 25 μg/mL concentration, that was used as supposed maximal nisin concentration on the surface. The antimicrobial activity was expressed as a diameter of microbial growth inhibition zone (*IZ*) around the sample well after the incubation, calculated as average of four independent measurements. Ageing effect, i.e., reduction of antibacterial activity (*R_A_*) of released nisin with time, was calculated as ratio of *IZ* diameter after given time from their preparation and *IZ* diameter of freshly prepared sample, following the Equation (1).

(1) $$R_{A}=\left(1-\frac{IZ_{t}}{IZ_{0}}\right)\times 100\;\left[\%\right]$$

in which *IZ_t_* is the diameter of inhibition zone in mm related to nisin released from stored sample and *IZ*_0_ is the diameter of inhibition zone in mm of nisin released from freshly prepared samples.

The antibacterial susceptibility testing of the nisin layer (surface) was covered by standards EN ISO 22196 *Measurement of antibacterial activity on plastics and other non-porous surfaces*. The specimens of untreated and treated blank material (without nisin) and untreated and treated material with nisin coating were used to measure viable cells after incubation immediately after inoculation and after incubation for 24 h. Prepared flat samples diameter was (25 ± 2) mm, and target concentration was 6 × 10^5^ cfu/mL. On the surface, 0.4 mL of test inoculum was pipetted; then, it was covered with the sterile foil and gently pressed down. The Soybean casein digest broth with lecithin and polyoxyethylene sorbitan monooleate (SCDLP) broth was used for washing of specimens.

For each test specimen, the number of viable bacteria recovered was determined in accordance with Equation (2):

(2) $$N=\frac{\left(100\times C\times D\times V\right)}{A}$$

in which *N* is the number of viable bacteria recovered per cm^2^ per test specimen; *C* is the average plate count for the duplicate plates; *D* is the dilution factor for the plates counted; *V* is the volume, in mL, of SCDLP added to the specimen; and *A* is the surface area, in mm^2^, of the cover film

Antibacterial activity (bacteria reduction) was calculated as according to Equation (3)

(3) $$R=U_{t}-A_{t}$$

in which *R* is the antibacterial activity; *U_t_* is the average of the common logarithm of the number of viable bacteria, in cfu/cm^2^, recovered from the blank test specimens after 24 h; and *A_t_* is the average of the common logarithm of the number of viable bacteria, in cfu/cm^2^, recovered from the treated test specimens after 24 h.

### 2.6. Surface Characterization

#### 2.6.1. Wettability and Surface Energy Analysis

Contact angle measurements of PVA films (nisin coated, uncoated, plasma treated and untreated) were performed using Surface Energy Evaluation system (See System E, Advex instruments s.r.o., Brno, Czech Republic). Demineralized water and diiodomethane were used as reference liquids. The analysis of a drop (volume of each drop was 10 µL) on a film surface was done after 20 s in order to let the drop stabilize its shape. Each sample was measured five times, mean contact angle was calculated, and subsequently the solid, surface-free energy was determined. The total surface-free energies *γ^total^* were calculated according to Owens approach that assumes that total surface-free energy is a sum of both dispersion and polar components, i.e., Lifschitz-van der Waals and electron acceptor/electron donor [19,21,22]. The results presented further are the average values from at least five measurements. Standard deviation value was always up to 10% of average value. 

#### 2.6.2. Elektrokinetic Characterization

Zeta potential of all samples was determined by SurPASS Instrument (Anton Paar-SurPass, Graz, Austria). The SurPASS instrument is an electrokinetic analyzer utilized for the investigation of the zeta-potential of macroscopic solids based on the streaming potential and/or the streaming current method. For the sample analysis, the adjustable gap cell was used in contact with the 1 mmol/dm^3^ KCl electrolyte. All measurements were performed at laboratory temperature.

The samples with higher PVA crosslinking degree of 10%, 20%, and 40% were analyzed in pH range of 4.5 to 9. The not-crosslinked and low crosslinked (PVA crosslinking degree 5%) samples were not stable, so they could be analyzed only at one pH value. For each measurement, a pair of identical samples (prepared on PE foils) was fixed on two opposite sample holders (with a cross section of 20 × 10 mm^2^ and gap between them of 100 µm). The instrument employs Smoluchowski’s equation [44] to determine the electrophoretic mobility of the particle and subsequently convert it in the Zeta Potential (see Equation (4)).

(4) $$\zeta =\frac{µ\sigma }{\varepsilon \varepsilon_{0}}\frac{\Delta V}{\Delta p}$$

in which *µ* is viscosity; *σ* conductivity; *ε*, *ε*_0_ permittivity; and linear fits to the pressure-voltage curve give zeta potential.

#### 2.6.3. XPS Analysis

The XPS measurements were done using the ESCALAB 250Xi (ThermoFisher Scientific, Waltham, MA, USA) system equipped with 500 mm Rowland circle monochromator with microfocused Al Kα X-Ray source. An X-ray beam with 200 W power (650 microns spot size) was used. The survey spectra were acquired with pass energy of 50 eV and resolution of 1 eV. High-resolution scans were acquired with pass energy of 20 eV and resolution of 0.1 eV. In order to compensate the charges on the surface, an electron flood gun was used. Spectra were referenced to the hydrocarbon type C 1s component set at a binding energy of 284.8 eV. The spectra calibration, processing, and fitting routines were done using Avantage software (ThermoFisher Scientific, Waltham, MA, USA).

## 3. Results

### 3.1. Stability Analysis of Nisin

As it is well known, nisin is very unstable in pure form, without any supplements as a stabilizer. This fact can cause difficulties during the monitoring of its release and adhesion. For this reason, the additional techniques were used to verify the nisin stability and nisin presence due to total nitrogen content measurement.

Electrophoretic assay and subsequent peptide determination (according to Bradford method) [40,41,45] were performed to evaluate nisin stability. The results from Bradford assay were compared to the total nitrogen content (TN) measurement, and it was found that amount of nitrogen corresponds to the nisin decomposition products—amino acids and shorter peptides—that are present in the immersion solution.

Based on the release behavior, it was also estimated that nisin was attached to the surface.

The mean molar weight of the peptide mixture (from electrophoretic measurements) confirmed that the stability of nisin attached to the PVA surface is dependent on the cross-linking degree and on the presence or absence of the plasma treatment step. These results were also obtained from TN measurements. Nisin molecule (C_143_H_230_N_42_O_37_S_7_) contains about 17% of nitrogen. Detected nitrogen (correlated to blank) was much higher than it would be if it corresponded to stable nisin measured by electrophoresis and HPLC (Table 1). This fact signifies that nisin undergoes decomposition, and nitrogen comes from present amino acids. Thus, amount of nisin that could be attached to the PVA surface was estimated from complementary methods. As it was confirmed later, by using antimicrobial activity testing and zeta potential measurement, the most stable (ageing resistant) nisin layer was observed at 20% PVA crosslinking. When 10% crosslinking was used, the nisin attachment (initial amount of nisin at the surface) was maximal, but the stability was lower than in the case of 20% crosslinking. On the surface of films with 40% crosslinking, the relative small amount of nisin was attached, and also its instability was observed. However, the antimicrobial activity testing (see Chapter 3.3.) of this film is still relatively high compared to estimated nisin concentration, which can be attributed to glutaric acid (crosslinker) activity [39,45].

### 3.2. Nisin Release

The nisin release profile was monitored, after its adhesion on the surface, in comparison to the study [45], in which nisin release from PVA bulk was investigated. 

**Figure 1:**
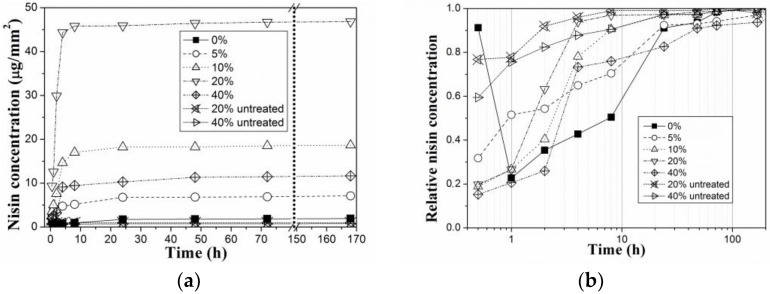
Nisin release from the PVA films after immersion in physiological solution, analysed by RP-HPLC: (**a**) cumulative concentration in µg/mm^2^ and (**b**) related to total released amount.

The effect of PVA crosslinking degree on amount of nisin released and probably also adsorbed on PVA substrate can be clearly recognized in the release study presented in Figure 1. It is evident that crosslinking degree of PVA influences extent of nisin adsorption, as well as its release profile. Untreated PVA films reveal only minimum nisin adsorption. Then, apparently, nisin is released immediately after immersion in salt solution (it means after 1–24 h). It was found that nisin was strongly attached at the films of 10% and 20% crosslinking degree. Nisin was released slowly in small amounts. On the other hand, from the film with 0% or 5% crosslinking the whole attached amount of nisin was released immediately after immersion, as it was in the case of untreated films. Besides, PVA with low crosslinking dissolved very fast. Pure PVA (without crosslinking) and also untreated PVA films do not reach the corresponding nisin attachment. The highest nisin surface adsorption was found for the samples with 20% of crosslinking degree. But for 40% crosslinking degree, the nisin adhesion is unsatisfying. This could be explained by the change in physical properties of PVA. It was proved also in some previous study that increasing the crosslinking degree leads to a significant decrease of the solubility. Moreover, PVA with 20% of crosslinking degree exhibited more advantageous mechanical properties for further applications [45]. Additionally, plasma treatment was not very effective in this case. It means that more intensive initial introducing of crosslinking connections leads to apparent change of material properties and formations of inhomogeneity on the surface that are caused by higher inhomogeneity in the bulk, higher degree of crystallinity, inhomogeneous formation of crystalline phase of glutaric acid.

### 3.3. Antimicrobial Assays

**Figure 2:**
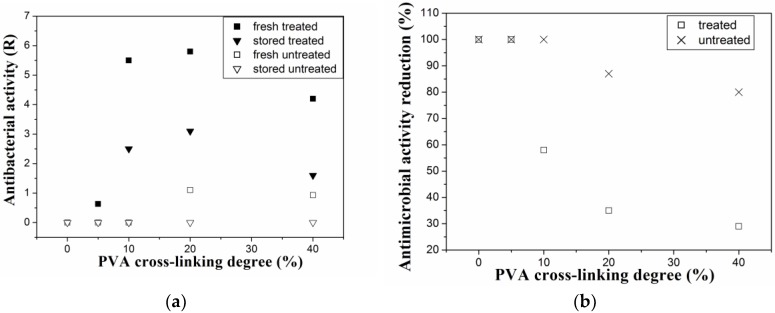
Antibacterial activity change of PVA films with attached nisin after 90 days of storage (**a**) after immersion in water solution for 3 h and (**b**) by ISO 22196 against *Staphylococcus aureus* (CCM 4516).

Anti-staphylococcal effectivity of nisin-coated PVA film was evaluated by antimicrobial susceptibility testing, with results shown in Figure 2a,b. Nisin in the PVA surface layer can be active against Gram positive (G+) bacterial strain *Staphylococcus aureus* even after 90 days of storage. However, nisin activity (and its ageing) is strongly dependent on the crosslinking degree (amount of GA) and on presence or absence of the plasma treatment step. The amount of GA influences not only bulk but also surface properties, and therefore the plasma treatment effectiveness. Only 35% reduction of antimicrobial activity after 90 days was observed in the case of nisin-coated PVA samples with 20% crosslinking degree, while almost 100% reduction of antimicrobial activity was observed in the case of 0% and 5% of crosslinking. It should be mentioned that synergic effect of the crosslinking agent, GA, cannot be neglected. Mainly in the samples with 40% crosslinking degree, modest antibacterial activity of GA in the systems has been found. However, this factor does not represent significant contribution to antibacterial activity of the PVA/GA/nisin samples [17,45]. Therefore, nisin is be supposed to be the principal antibacterial agent against G+ bacterial strains in the studied systems.

To compare it to HPLC analysis (Figure 1b), testing of released nisin was also performed by MHA diffusion testing, which is dependent on migration rate of antimicrobial agent and on its stability (Figure 2a). Antibacterial activity of released nisin from films that was stored at specific conditions for 90 days was determined according to inhibition zones of nisin in immersion solution.

Antimicrobial properties of PVA films depend on the amount of nisin that is attached to the PVA film and also on the nisin release from the PVA surface so the main aim should be to ensure maximal initial activity (surface concentration) of nisin and its stability in the layer. As was shown in antimicrobial testing, the crosslinking degree has an important influence on nisin adhesion.

It was not possible to calculate exact amount of nisin attached on the surface, but this amount can be estimated from the amount that is released from the surface.

Nisin layers were tested as promising natural coating with anti-staphylococcal activity [6,9,11,17,18], and it was proved that even in combination with PVA surface, it can improve the antimicrobial activity of biocompatible films. Nevertheless, the key role lies in the plasma treatment, as was confirmed for other polymer materials in literature [16,19,22,29]. This corresponds also to our finding, when plasma treatment of our samples led to obtaining an effective layer with suitable nisin adhesion.

### 3.4. Wettability and Surface Energy Analysis

**Figure 3:**
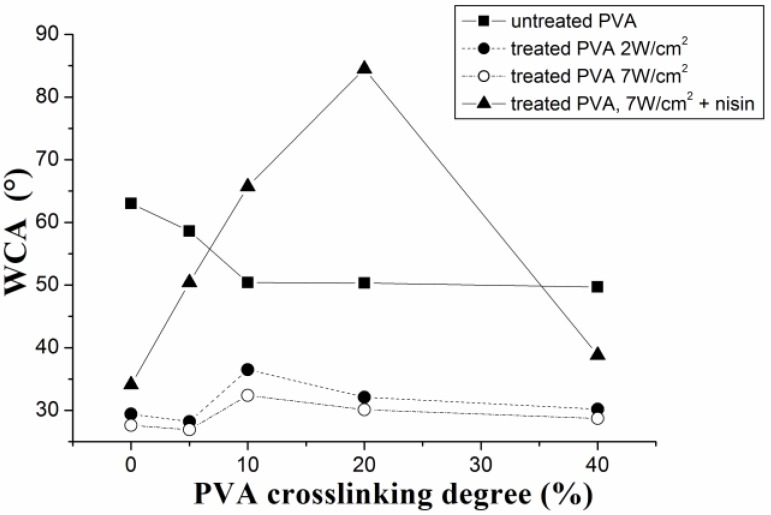
The water contact angle dependence as a function of PVA crosslinking degree; plasma treatment time was 10 s. Nisin was attached from PBS buffer.

As it is shown in Figure 3, the PVA plasma treatment using the 2 W/cm^2^ or 7 W/cm^2^ power density resulted in essentially the same wettability. Nevertheless, the 7 W/cm^2^ was finally chosen for the rest of experiments, since it was found that a decrease in water contact angle (WCA) of plasma-treated PVA films was sufficient for PVA wettability sustainment (WCA decrease is higher than 50% of the initial value).

**Figure 4:**
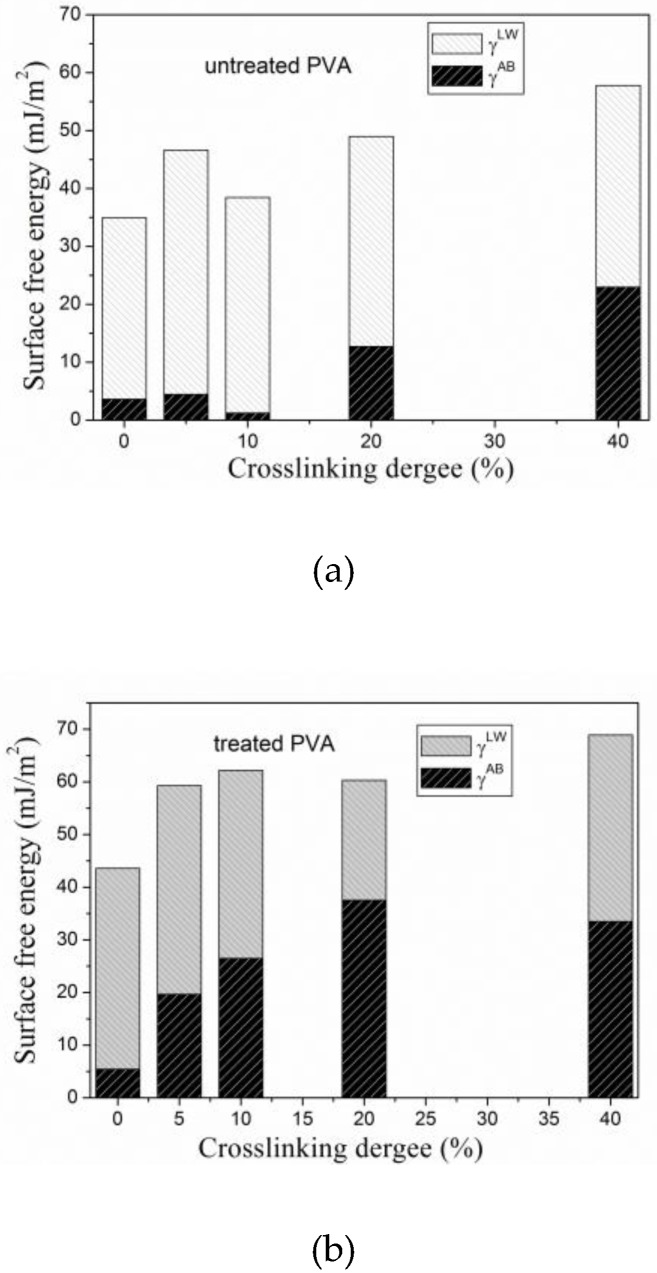

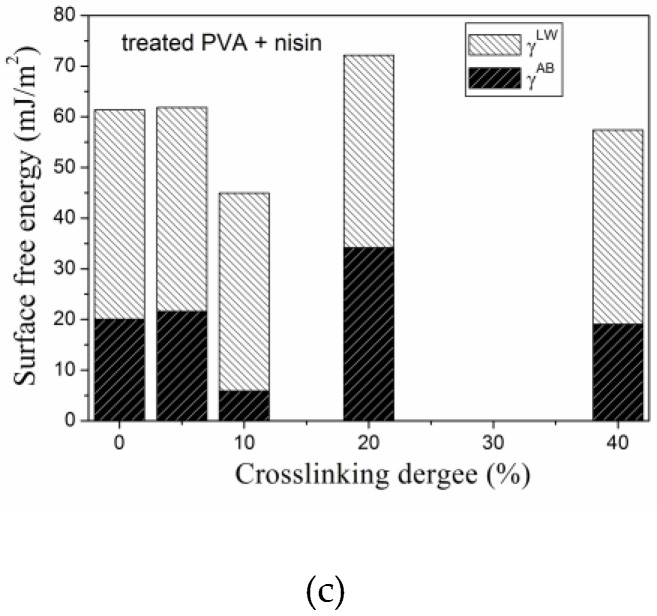
Surface-free energy evaluation for (**a**) untreated, (**b**) plasma-treated PVA films, and (**c**) films with attached nisin.

Initial concentration of nisin (that was measured during nisin release) corresponded to nisin adhesion on PVA films, and it is in agreement with WCA measurements (Figure 4). The hydrophilic character (wettability) of PVA films significantly increased after the plasma treatment (see Figure 3 and Figure 4). The *γ^AB^* component of surface-free energy increases with plasma treatment, which indicates the presence of polar groups (attached via covalent bonds). Furthermore, the total surface energy increases, which enables nisin adhesion. With nisin immobilization on the surface, the *γ^LW^* increases, thus the van der Waals interactions predominate. The different trend (WCA increase) with nisin adhesion demonstrates increase of contact angle, suggesting the hydrophobic nisin groups’ attendance. While non-crosslinked PVA films and films with low crosslinking degree up to 5% show only slightly decreased wettability in comparison with untreated PVA films, samples with higher crosslinking degree (10 and 20%) show significant increase in WCA. This increase can be also caused by the surface roughness. With further increasing of crosslinking degree, the steep decrease of WCA was observed. This phenomenon can be explained by lower presence of free hydrophilic groups (OH, COO–) on the PVA surface, as was also confirmed by the previous study [45]. Maximal WCA of 84° was obtained for PVA with 20% of crosslinking. This can be attributed to protein surface adhesion when nisin was bonded to PVA substrate. It also reveals the optimal PVA crosslinking degree for the protein efficient surface immobilization. These results are in agreement with those in Section 3.1 and Section 3.2.

### 3.5. Electrokinetic Characterization

The stability study of coatings can be carried out, too, in correlation with the electrokinetic measurements. The surface charge on material is related to affinity-associated interactions. It can control degree of biomolecular affinity on a polymer surface [43,46,47,48,49,50]. Zeta potential is used for characterization of natural and synthetic polymers and in investigation of their hydrophilicity, which is important for their interactions with biomolecules, dyes, surfactants, etc. [30,31,42,44,50]. It is also important for research of modified surfaces (such as membranes, filters) or polymers modified by a plasma treatment or by plasma chemical coatings [32,50]. Water soluble polymers interact according to their functional group. Zeta potential of biodegradable polymer surface is not often evaluated in literature. In this paper, zeta potential, combined with pH of liquid phase, was used to characterize electrostatic interactions between the polymer surface and the immobilized bioactive peptide.

**Figure 5:**
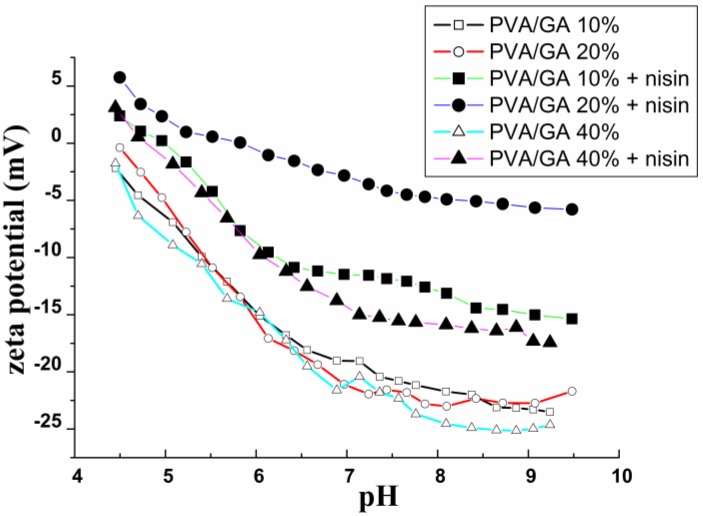
Zeta potential of treated PVA films with different crosslinking degree with and without adsorbed nisin as a function of pH values. Titration was performed by using 0.05 M NaOH. Non-crosslinked films and films with crosslinking up to 5% were not measured due to their low resistance to electrolyte.

The zeta-potential measurement provides complementary information to WCA and surface-free energy evaluation. Results for zeta potential of treated and untreated films, and for the films with attached nisin, are shown in Figure 5. As it was expected, the zeta potential value increased after the plasma treatment (shift to more negative values). This behavior suggests that the surface is not stable; thus, it is susceptible to nisin attachment. Zeta potential of PVA films without nisin presents negative values in all cases. Lower (more negative) values were recorded in the case of the crosslinked PVA, mainly with higher content of glutaric acid (higher crosslinking degree). As zeta potential values are included inside the domain ± 30 mV, it means that the stability of measured surface is reduced; it means that the zeta potential measurements indicate the possibility of nisin adhesion. Surface charge becomes positive or less negative after nisin adhesion on PVA. Even the zeta potential values are dependent also on the surface roughness; it is clear that plasma-treated PVA show less negative zeta potential. Thus, it is confirmed that plasma treatment enhances the potential of nisin adsorption. In samples with nisin content, the shift to higher values was observed, reaching 5 mV at pH 4.5 in case of 20% of crosslinking degree. Moreover, as can be seen in Figure 5, the titration curve for PVA with 20% crosslinking shows the shift to the value close to zero at pH of 8 (pH of nisin is 8.3–8.5). All these findings signify the higher nisin adsorption and stability. The titration curves show the film high instability when the nisin is bonded, especially in the case of film with 10% of crosslinking.

### 3.6. XPS Analysis

Nisin attachment chemistry was estimated by XPS measurements. Surface composition of untreated and plasma treated samples is presented in Table 2. PVA films’ ability to adsorb nisin after plasma treatment was investigated by analyzing changes in O and C peaks on the treated and untreated PVA films (PVA without crosslinking agent was used). The strong increase of O peak was observed on the surface of plasma treated samples (see Table 2).

**Figure 6:**
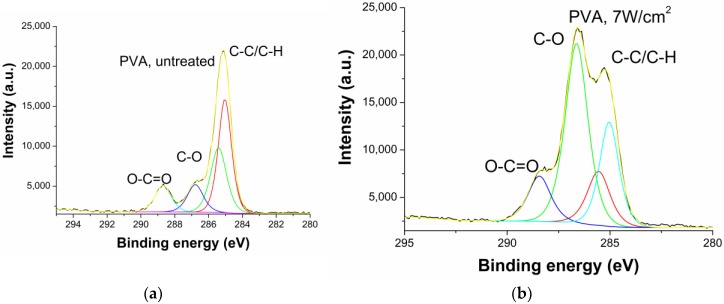
C1s high resolution peak fitting of (**a**) untreated PVA (0% crosslinking) and (**b**) plasma treated PVA (0% crosslinking).

The peak fitting routines were done in order to evaluate the changes in the bond structure and, therefore, explain the observed changes after plasma treatment. The high resolution C 1s peak was fitted with 6 principal components: C–C/C–H (binding energy at 284.7 eV), C–N (285.6 eV), C–O (286,3 eV), N–C–O (287.6 eV), C=O (288.1 eV), and O–C=O (289.2), as it is shown in Figure 6. The results of the fits are presented in Table 3.

As it was mentioned above, a significant decrease in WCA was observed after the plasma treatment. Hydrophilic character of PVA surface was achieved, because characteristic polar functional groups (mainly COO– groups) were introduced onto the PVA surface by the plasma treatment [10,13] (see Figure 6 and Table 3).

The C1s peak fitting of plasma-treated samples shows O–C=O arising that can be attributed to the polar functional group formation. After nisin attachment, also C=O, C–O vs. C–C bonds presence was evaluated. Results (Table 3) show decrease of C–O and C–H bonds and appearance of N–C=O and O–C=O after nisin adhesion, while after final plasma treatment, the C–O and N–C=O bonds presence is higher. This can be caused by nisin attachment. On the other hand, the O–C=O are not present.

## 4. Conclusions

Combination of coplanar Surface Barrier Discharge activation and cross-linking using environmentally friendly glutaric acid was applied to PVA to study its potential use as a carrier system for nisin adhesion. It was found that both mentioned factors have a crucial effect on the extent of nisin surface attachment and its release kinetics. 

It was proved that crosslinking degree of polyvinyl alcohol plays an important role mainly in the range of 10–20%, which was evaluated as optimal for preparation of relatively water-stable peptide coatings that can sustain also the antibacterial properties with potential applicability in the medical field. According to the zeta potential measurement, the adhesion proceeded at acidic pH value, and surface inhomogeneity was observed, mainly in the case of 10% of crosslinking. This suggests that besides the bulk, also the surface properties of the polymer materials must be carefully controlled to achieve acceptable results.

Based on the results, DCSBD plasma treatment of PVA using 20% crosslinking with glutaric acid and nisin adhesion represents a novel and promising method for the design of biomaterials characterized by tunable stability and diffusion of bioactive compounds.

## Figures and Tables

**Table 1:** Attached nisin content (Bradford assay from electrophoresis) and nitrogen content (TN) on the PVA surface (PVA films were stored at 25 °C for 3 months, relative humidity 54%). Nisin content on untreated PVA films was not measurable, and SD was higher than calculated value.

PVA/Nisin-Buffer Crosslinking Degree (%)	Nisin Content (µg/mm^2^)	TN (µg/mm^2^)
0	1.8 ± 0.5	0.7
5	3.6 ± 1.5	4.3
10	31.4 ± 1.5	13.2
20	28.2 ± 1.5	9.7
40	19.3 ± 1.3	6.8

**Table 2:** XPS elemental analysis of PVA samples.

PVA + 0% GA	C (at %)	O (at %)
untreated	68	32
treated 2 W/cm^2^, 10 s	38	45
treated 7 W/cm^2^, 10 s	30	51

**Table 3:** Areas of C1s components.

Bonds (%)	Untreated	Treated, 7 W/cm^2^	Treated with Nisin
C–C/C–H	41	31	21
C–N	34	36	19
C–O	13	15	47
C=O	12	2	2
N–C=O	0	5	11
O–C=O	0	11	0
